# Combined Immunotherapy With Belatacept and BTLA Overexpression Attenuates Acute Rejection Following Kidney Transplantation

**DOI:** 10.3389/fimmu.2021.618737

**Published:** 2021-02-24

**Authors:** Hengcheng Zhang, Zijie Wang, Jiayi Zhang, Zeping Gui, Zhijian Han, Jun Tao, Hao Chen, Li Sun, Shuang Fei, Haiwei Yang, Ruoyun Tan, Anil Chandraker, Min Gu

**Affiliations:** ^1^ Department of Urology, The First Affiliated Hospital of Nanjing Medical University, Nanjing, China; ^2^ Transplantation Research Center, Renal Division, Brigham and Women’s Hospital, Harvard Medical School, Boston, MA, United States

**Keywords:** kidney transplantation, Belatacept, acute rejection, BTLA, CTLA-4, immunosuppressant

## Abstract

**Background:**

Costimulatory blockade provides new therapeutic opportunities for ensuring the long-term survival of kidney grafts. The adoption of the novel immunosuppressant Belatacept has been limited, partly due to concerns regarding higher rates and grades of acute rejection in clinical trials. In this study, we hypothesized that a combined therapy, Belatacept combined with BTLA overexpression, may effectively attenuate acute rejection after kidney transplantation.

**Materials and Methods:**

The rat kidney transplantation model was used to investigate graft rejection in single and combined therapy. Graft function was analyzed by detecting serum creatinine. Pathological staining was used to observe histological changes in grafts. The expression of T cells was observed by immunohistochemistry and flow cytometry. *In vitro*, we constructed an antigen-stimulated immune response by mixed lymphocyte culture, treated with or without Belatacept and BTLA-overexpression adenovirus, to observe the proliferation of receptor cells and the expression of cytokines. In addition, western blot and qRT-PCR analyses were performed to evaluate the expression of CTLA-4 and BTLA at various time points during the immune response.

**Results:**

In rat models, combined therapy reduced the serum creatinine levels and prolonged graft survival compared to single therapy and control groups. Mixed acute rejection was shown in the allogeneic group and inhibited by combination treatment. Belatacept reduced the production of DSA and the deposition of C4d in grafts. Belatacept combined with BTLA overexpression downregulated the secretion of IL-2 and IFN-γ, as well as increasing IL-4 and IL-10 expression. We also found that Belatacept combined with BTLA overexpression inhibited the proliferation of spleen lymphocytes. The duration of the elevated expression levels of CTLA-4 and BTLA differentially affected the immune response.

**Conclusion:**

Belatacept combined with BTLA overexpression attenuated acute rejection after kidney transplantation and prolonged kidney graft survival, which suggests a new approach for the optimization of early immunosuppression after kidney transplantation.

## Introduction

Compared to classic maintenance dialysis, kidney transplantation is considered to be an optimal treatment option with improvement in life quality and prolongation of survival in end-stage renal disease patients (1, 2). In recent years, along with the amelioration of surgical techniques, popularization of organization matching and application of new immunosuppressants, apparent improvement has been observed in short-term graft survival among recipients (3). Nevertheless, ensuring the long-term survival of kidneys after transplantation remains an important objective that involves the consideration of a number of immunological and nonimmunological factors, especially acute rejection (4, 5). Therefore, immunotherapy against acute rejection still attracts considerable attention in the clinic (6).

Acute rejection occurs most commonly within three months after transplantation and clinically results in decreased urine, increased serum creatinine, swelling and pain of graft (7). Based on pathology, there are two types of acute kidney allograft rejection: T-Cell Mediated Rejection (TCMR) and Antibody-Mediated Rejection (ABMR). TCMR is caused by the immune response between T cells and antigens present in recipient transplanted kidneys (8), with tubulitis, interstitial inflammation and especially intimal arteritis as a feature. In addition, antigen-specific binding of receptor circulating antibody to endothelial cell allografts leads to ABMR, which is characterized by the histologic evidence of acute tissue injury including glomerulitis and peritubular capillaritis as well as the existence of circulating donor specific antibody (DSA) and deposition of complement degradation products C4d in grafts (9). Immunosuppressive regimens for acute rejection therapy have been the focus of transplant research. However, increasing clinical concerns have occurred with nephrotoxicity and negative effects due to the excessive immunosuppression of current immunosuppressants such as calcineurin inhibitors (CNIs) and negative effects of overimmunosuppression (10, 11). Furthermore, effective treatments are still lacking in acute ABMR, correlated with severe clinical symptoms and poor prognosis (12). Consequently, there remains a need for further optimized therapeutic strategies to prevent allograft rejection (13).

Costimulatory and coinhibitory pathways, which are the second signals of T cell activation, play an essential role in transplantation immunity. Several research studies have indicated that coinhibitory molecules, such as PD-1 and CTLA-4, can transmit negative signals by attenuating T cell activation, inhibiting cell proliferation and inducing immune tolerance (14). Enhancing coinhibitory signals has potential clinical application value in transplantation immune-regulation and rejection inhibition (15). Belatacept (CTLA-4 fusion protein) suppresses T cell activation *via* competitively blocking the binding of the antigen-presenting cell (APC) surface molecules CD80\CD86 to CD28 and has been approved by the FDA against acute rejection (AR) in renal transplant recipients in 2011 (16). Retrospective studies reported a lower risk of hypertension and cardiovascular disease, similar graft survival, as well as sustained improvement in renal function following treatment with Belatacept when compared to CNI-treated patients (17–19). Additionally, Belatacept showed remarkable benefits in recipients with CNI intolerance or chronic allograft nephropathy (20). However, the high incidence of acute cellular rejection after surgery is one of its limitations, and the rate of AR in the Belatacept group was up to 24% at three years in the clinical BENEFIT trial. Infection risk, urinary tract infections and cytomegalovirus infections were most common, and adverse reactions of maintenance dose, such as posttransplant lymphoproliferative disorder, cannot be ignored (21, 22). In fact, the high-cost burden is another obstacle for Belatacept popularization. Therefore, the routine clinical application of Belatacept is controversial, especially in recipients diagnosed with AR.

A newly found coinhibitory molecule, BTLA is expressed widely in innate and adaptive immunocytes and increases expression when activated (23). The inhibitory effect of BTLA on the immune response has been confirmed by recent studies; for instance, targeted BTLA therapy can inhibit rejection in a mouse heart transplantation model (24) but less so in kidney transplantation. Our previous research has shown that the BTLA pathways were involved in the pathogenesis of AR in biopsy-proven recipients following kidney transplantation, and BTLA overexpression can suppress TCMR by regulating T cell receptor downstream signals (25, 26). Additionally, several studies indicated the potential value of combining costimulatory or costimulatory molecules in disease treatment, which may reduce adverse effects through a lower single dose and provides new ideas for the prevention of kidney transplant rejection (27). Based on these findings, we speculated that Belatacept combined with the BTLA pathway can ameliorate the occurrence of acute rejection following kidney transplantation, inhibit T cell activation and proliferation in recipients, improve kidney graft functions and prolong graft survival. This study investigated this hypothesis by using a rat renal transplant model of acute rejection and mixed lymphocyte reaction *in vitro* experiments.

## Materials and Methods

### Ethics Statement

All animal studies were strictly performed following the Nanjing Medical University Animal Care and Use Committee guideline (Ethical Approval Number: IACUC1601140-1).

### Animals and Reagents

Major histocompatibility complex (MHC) fully mismatched SD and Wistar rats were purchased from Charles River Laboratory (Beijing, China). Belatacept was obtained from Bristol-Myers Squibb (NY, USA), and BTLA overexpression adenovirus and negative-control vectors (CMV-MCS-3FLAG-SV40-EGFP, which is a linear double-stranded DNA virus with a wide host range and the ability to infect dividing and non-dividing cells) were constructed by Genechem (Shanghai, China). Anti-CTLA-4 (Santa-Cruz, USA), anti-BTLA (Abbiotec, USA), anti-GAPDH (Abcam, USA), anti-CD3 (Abcam, USA) and anti-Foxp3 (Abcam, USA) antibodies were used for Western blot or immunohistochemistry (IHC) staining. Anti-C4d (American Research Products, USA) and anti-CD138 (Abcam, USA) antibodies were obtained for immunofluorescence staining. We obtained the flow antibodies APC-labeled anti-CD3, FITC-labeled anti-CD4 and PerCP-eFluor710-labeled anti-CD8 from eBioscience (CA, USA). We used rat GM-CSF, IL-4 and TNF-α (Prospec-Tany, ISR) to stimulate dendritic cells (DC).

### Kidney Transplantation Model

Rat kidney transplantations were carried out according to a previously described procedure (26). Two experienced microsurgeons performed the surgeries in a sterile environment. In brief, we separated and removed the donor rat left kidney and ureter and then transplanted it to the left renal fossa of recipients that underwent bilateral nephrectomy. The renal arteriovenous was anastomosed end-to-end, and the ureter was embedded into the recipient’s bladder. The renal artery was seen to pulsate, the ureter engorged, and the graft returned to a ruddy complexion. The whole surgical procedure was completed in 2 h with an anastomotic time of approximately 40 min. The surgery-related data for each group are shown in **Table S1**.

Rats were randomly divided into different treatment groups (n=5 for each group and time-point). Kidneys from SD rats transplanted into SD rats constituted the Syngeneic (Syn) group. Wistar rats were donors with the SD rats as recipients in the Allogeneic (Allo) group to induce acute rejection. The other groups consisted of (1) the Allo+Control group: the allogeneic recipients were pretreated negative-control vectors two days before surgery; (2) the Allo+BEL group: Belatacept (60 mg/kg) was injected into SD rat abdomens at postoperative and 4 days after transplantation; (3) for the Allo+BTLA-Over group, the SD recipients were preinjected with BTLA overexpression adenovirus (1×109 PFU/each) two days posttransplant; and (4) for the Allo+Combination group, the recipients were pretreated with BTLA adenovirus and administered Belatacept (**Figure S1**). The recipients were harvested for graft tissue and blood at each time point. Additionally, eight recipients in each group were observed for graft survival. We observed postoperatively the urine output of recipients, and anuria was considered the end of kidney graft survival (28).

### Histopathology Examination

The detailed procedure has been described previously (26). The harvested grafts were placed into 10% buffered formalin and then stored in paraffin. These tissues embedded were sectioned at a 4 µm thickness. Hematoxylin and eosin (HE) staining was done in accordance with standard techniques. Pathological manifestations of acute rejection were evaluated based on Banff 2017 classification (29)

### Immunohistochemistry and Immunofluorescence Staining

Immunohistochemistry was performed to detect the expression of CD3, CTLA-4 and BTLA in graft and Foxp3 in the spleen of recipients. The operation method refers to the standard protocol. Isotype control using a non-immune antibody of the same isotype and at the same concentration as the primary antibody was performed as staining control. Then, we took 8 high-resolution images under the microscope and used image pro plus5.0 software to measure the relative expression of the target protein with integrated optical density (IOD). We used the graft tissue sections with anti-C4d (dilution: 1:50) and anti-CD138 (dilution: 1:500) antibodies and corresponding secondary antibodies to do immunofluorescence staining. The fluorescence intensity per unit area was used to analyze the relative expression in the kidney.

### Serum Creatinine Detection

To analyze the change in renal function of the recipient rats, we used a creatinine detection kit (Jiancheng BI, China) to detect the serum creatinine level according to the manufacturer’s instructions.

### Mixed Lymphocyte Reaction

In this study, primary SD rat spleen lymphocytes were used as immune responder cells in a mixed lymphocyte reaction. We extracted dendritic cells from the peripheral blood of Wistar rats and then cultured them in complete medium (containing 1640 medium, FBS, 50 ng/ml Rat-GM-CSF, 10 ng/ml Rat-IL-10 and 20 ng/ml Rat-TNF-α) for 7 days, which allowed them to become mature dendritic cells (mDCs) and act as antigen-presenting cells. The mDCs were counted and then evenly spread in 12-well plates with 5×10^4^/well or in 96-well plates (2×10^5^/well). We pretreated these mDCs with mitomycin C (30 mg/ml) in serum-free medium for 20 min before admixture. As response cells, the extracted primary spleen lymphocytes were laid in 12-well plates (1×10^6^/well) and 96-well plates (2.5×10^5^/well) to form mixed lymphocyte reactions. Cultures were maintained in complete medium for the required times at 37°C in 5% CO2 in the air. The reaction system and other details about treatment are shown in the results section.

### Western Blot Analysis

The graft tissues and response cells of MLR were collected and lysed in RIPA buffer with protease and phosphatase inhibitors. We extracted the proteins of tissues and cells following previous work and then determined protein concentrations. Equal quality proteins underwent polyacrylamide gel electrophoresis, were separated on 10% SDS gels and transferred onto polyvinylidene fluoride (PVDF) membranes. After blocking and washing, these membranes were incubated with anti-CTLA-4, anti-BTLA and anti-GAPDH antibodies. The band intensity and volume were clarified to examine the relative expression. All experiments were repeated three times.

### Quantitative Reverse Transcription-PCR

Kidney grafts were submerged in RNAlater stabilization solution (Sigma, USA) for freezing. Total RNA of graft tissues and cells was extracted using RNA extraction kits (Tiangen, China) and reverse transcribed to cDNA by a PrimeScript RT Kit (Takara, Japan). Quantitative RT-PCR was carried out *via* the SYBR Green PCR kit (Takara, Japan) according to the manufacturer’s instructions. The 2-ΔΔCt method was adopted to analyze gene expression. The sequences used in our study are as follows:

CTLA-4: forward: 5’-AGTGACCCAACCTTCAGTGG-3’,
reverse: 5’-AAGCCCAACGTGTTCTTCAC-3’;
BTLA: forward: 5’-ATCCCAGATGCTACCAATGC-3’,
reverse: 5’-TTGGGAGTTTGTCCTGGAAC-3’;
GAPDH: forward: 5’-GGCCTTCCGTGTTCCTACC-3’,
reverse: 5’-CGCCTGCTTCACCACCTTC-3’. All experiments were repeated three times.

### Enzyme-Linked Immunosorbent Assay

We tested serum samples and supernatants of MLR by ELISA according to the instructions of the kits (Cusabio, China). At 450 nm, the OD values of each sample were measured to express the concentration of cytokines.

### Donor Specific Antibody Detection

We harvested spleens from donor Wistar and recipient SD rats as probes to detect DSA in serum. The fresh spleen cells were mashed through 70 µm filters, resuspended in PBS, and then added into 96-well plates with 5×10^5^/well after washed twice. The serum from kidney transplanted rats was diluted (1:50) and used to incubate the spleen cells for 30 min at room temperature. The cells were then washed and incubated with FITC-labeled anti-rat-IgG antibody (Jackson ImmunoResearch, PA, USA) for 30 min. DSA was measured by assessing anti-IgG signal by flow cytometry, with the incubated spleen cells from SD rats as staining control and expressed as the mean fluorescence intensity (MFI).

### Flow Cytometry

To explore the cell proliferation response in MLR, we used bromodeoxyuridine (BrdU) incorporation and then tested by flow cytometry. The protocol followed previous research. Additionally, peripheral blood of rat recipients at 7 days after transplantation was harvested and stained with APC-labeled anti-CD3, FITC-labeled anti-CD4 and PerCP-eFluor710-labeled anti-CD8 antibodies. Flow cytometry was determined by a Gallios flow cytometer and analyzed with FlowJo Software (Tree Star, OR).

### Statistical Analysis

Student’s t-test was used to compare the difference in expression levels among groups, and all data are expressed as the mean ± standard deviations (SD). To compare the differences in graft survival, we performed Kaplan-Meier survival analysis and a log-rank test to compare the graft survival among each group. P-values of less 0.05 were considered to be significant. GraphPad Prism, version 8.0 (GraphPad Software) was used for statistical analysis.

## Results

### Establishment and Identification of Rat Renal Transplantation Model With Acute Rejection

**Figure 1:**
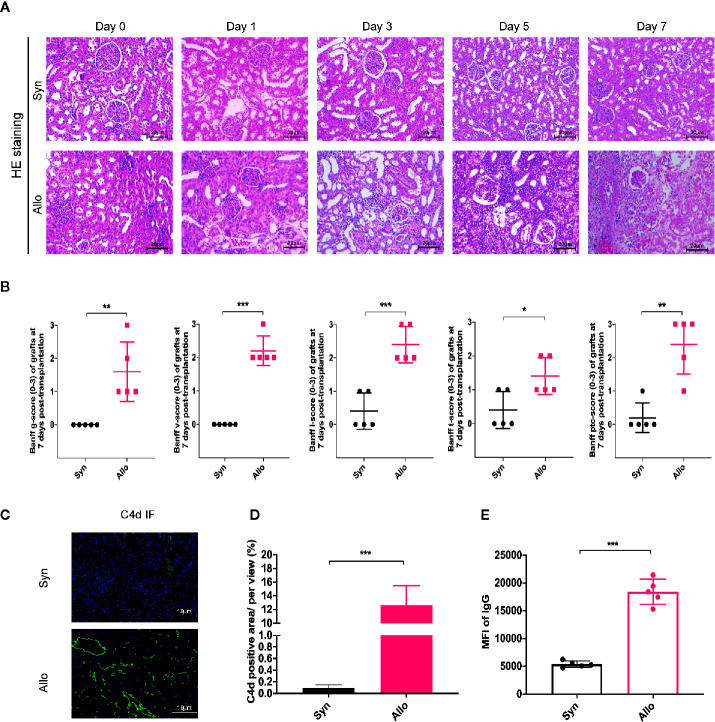
Construction of rat renal transplantation model with acute rejection. **(A)** Pathological staining analysis of kidney grafts from the Syngeneic group and the Allogeneic group recipients on preoperative Day 0 and postoperative Days 1, 3, 5 and 7 (Magnification: 200×). **(B)** Assessment of graft tissues glomerulitis (g), intimal arteritis (v), interstitial inflammation (i), tubulitis (t), and peritubular capillaritis (PTC) based on the Banff 2017 classification system. **(C)** Graft tissue-specific C4d immunofluorescence staining analysis on 7 days after transplantation (Magnification: 400×). **(D)** The proportion of C4d-positive regions was used to compare relative C4d-positive expression across groups. **(E)** Expression of serum DSA in each group were reflected by mean fluorescence intensity (MFI) of donor-related IgG in flow cytometry analysis. Results are expressed as mean ± SD, NS, no significant; *P < 0.05; **P < 0.01; ***P < 0.001.

To investigate the role and mechanism of the combination therapy on acute rejection *in vivo*, we established the acute rejection model of orthotopic kidney transplantation in rats with 5 recipients per timepoint in each group. The grafts were harvested for pathology analysis preoperatively and postoperative days 1, 3, 5 and 7. **Figure 1A** indicates that, in contrast to the preoperative state, the Syn group showed ischemia-reperfusion injury, renal tubular edema, and other mild acute renal injury caused by surgery on days 1 to 7, without acute rejection reactions. However, in grafts from the Allo group, we observed acute renal injury at day 1; subsequently, further manifestations with mononuclear cell infiltration, glomerulitis, and tubular injury could be seen from day 3 to day 5, which progressed to pathological mixed acute rejection characterized by moderate to severe intimal arteritis, glomerulitis, and peritubular capillaritis at day 7. Further statistical analysis based on Banff 2017 revealed a significant increase in all classification scores after allogeneic transplantation (**Figure 1B**). IF staining showed that linear C4d sedimentation in peritubular capillaries was increased in the Allo group compared to the Syn group. Serum donor specific antibody IgG was remarkably upregulated in the Allo group. (**Figures 1C–E**) Similarly, the Allo+Control group pretransfected by negative vector also exhibited progressive aggravating AR from day 3 to day 7, clear C4d sedimentation and IgG positivity at day 7 (**Figure S2**). Overall, classic pathological evidence of cell- and antibody-mediated rejections was present in the allogeneic group and was most pronounced at postoperative day 7, and there was no significant effect of negative vector intervention in the Allo+Control group.

### Combination Therapy Improved Renal Function and Prolonged Graft Survival

**Figure 2:**
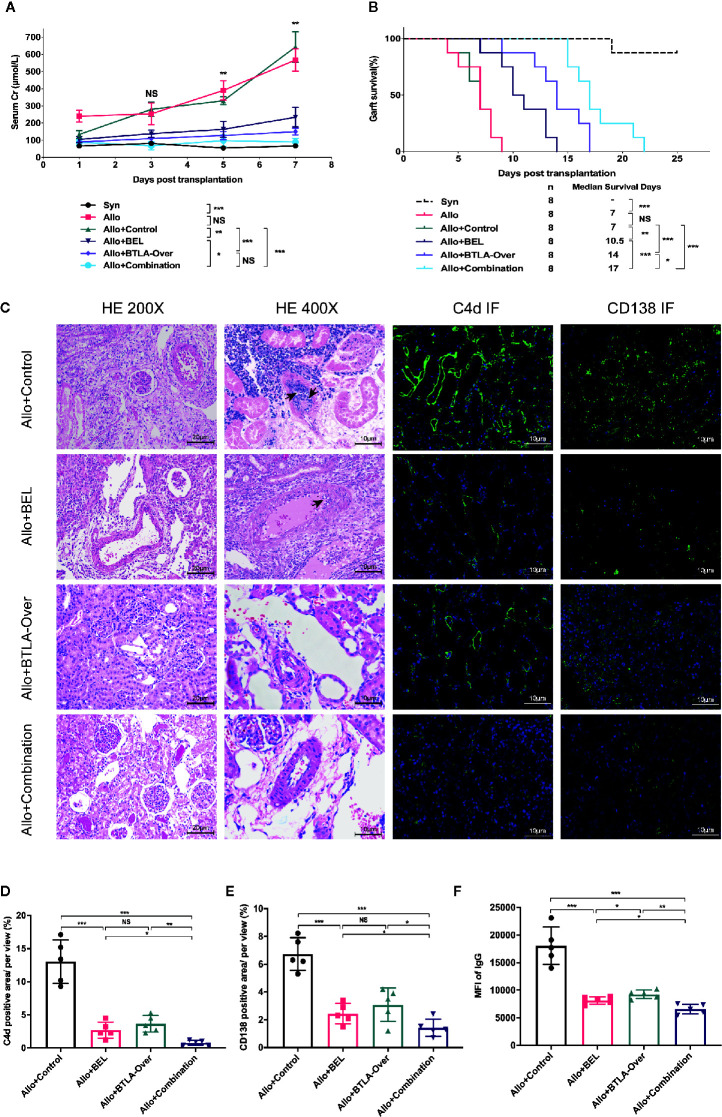
Combination therapy improved renal function, prolonged graft survival and effectively suppressed acute rejection after kidney transplantation. **(A)** Serum creatinine levels at various timepoints after kidney transplantation among each group of recipient rats. **(B)** Analysis of graft survival time posttransplantation of recipient rats. **(C)** Postoperative day 7, recipient kidney graft tissue-specific HE and immunofluorescence staining analysis. Magnification: HE for nephron and renal interstitium: 200×; HE for arterial intima: 400×, Arrows: intimal arteritis; C4d immunofluorescence staining: 400×; CD138 immunofluorescent staining, 400×. **(D)** The proportion of C4d-positive regions was used to compare relative C4d-positive expression across groups. **(E)** Percentage of CD138-positive regions used to express relative CD138 expression levels in tissues. **(F)** MFI of IgG in flow cytometry was used to reveal the expression of serum donor-specific antibody (DSA) in each group. NS, no significant; *P < 0.05; **P < 0.01; ***P < 0.001.

First, we confirmed the effectiveness of intravenous overexpression adenovirus in normal SD rats by IHC, western blot and qRT-PCR analyses (**Figure S3**). Then, serum samples were collected for creatinine testing after transplantation to reflect renal function changes. We found that the Scr in the Allo group was consistently increased after surgery, which was significantly different than the Syn group. Belatacept, BTLA overexpression and the combination therapy can inhibit creatinine increase after kidney transplantation compared to the Allo+Control group (**Figure 2A**). Notably, although there was no significant difference between the Allo+Combination group and the Allo+BTLA-Over group, the combined intervention resulted in lower Scr values with a decreasing trend.

Eight recipients per group were constructed to evaluate graft survival. In contrast to the long-term survival in the Syn group, the median survival time was 7 days in the Allo group, which was also similar to the Allo+Control group. Surprisingly, the combination therapy clearly prolonged graft survival to 17 days, which was superior to the survival obtained with single therapy (**Figure 2B**). These results show that combining Belatacept and BTLA overexpression attenuated creatinine elevation induced by acute rejection, improved postoperative graft renal function and significantly prolonged graft survival.

### Combination Therapy Effectively Suppressed Acute Rejection After Kidney Transplantation

To investigate the effect of single intervention versus combination therapy on acute rejection, we performed characteristic pathological staining and DSA detection on recipient specimens from each group at postoperative 7 days (**Figure 2C**). What stands out in this figure is the clear decrease of glomerulitis and peritubular capillaritis in the Allo+BEL group compared with the Allo+Control group, while mild to moderate intimal arteritis was rarely seen under 400× microscopy. Only mild interstitial inflammation, tubulitis, and glomerular edema were shown in the BTLA overexpressed and combination treatment group without apparent cell-mediated rejection, such as intimal arteritis in the high-fold field.

Positive detection of C4d in peritubular capillaries and DSA in serum are features of ABMR. Further studies reported that C4d in graft IF staining were notably reduced in the Belatacept treatment group, as well as in the Allo+Combination group, compared to the Allo+Control group (**Figures 2C, D**). In addition, flow cytometry analysis revealed that serum DSA was particularly inhibited in the Allo+Combination group, rather than the Allo+Control group, which was caused by the reduction of antibody-producing CD138-positive plasma cells in grafts with combination therapy (**Figures 2C–F**). An additional interesting result that emerged was a more effective downregulation of CD138 infiltration and DSA production with Belatacept than single BTLA overexpression treatment. These findings indicate that Belatacept has not only a limited effect on TCMR but also a significant inhibitory effect on ABMR; overexpression of BTLA can obviously attenuate TCMR characterized by arterial endarteritis, and combination treatment can significantly inhibit mixed acute rejection.

### Combined Belatacept and BTLA Overexpression Affected T Cell Frequency in Recipients

**Figure 3:**
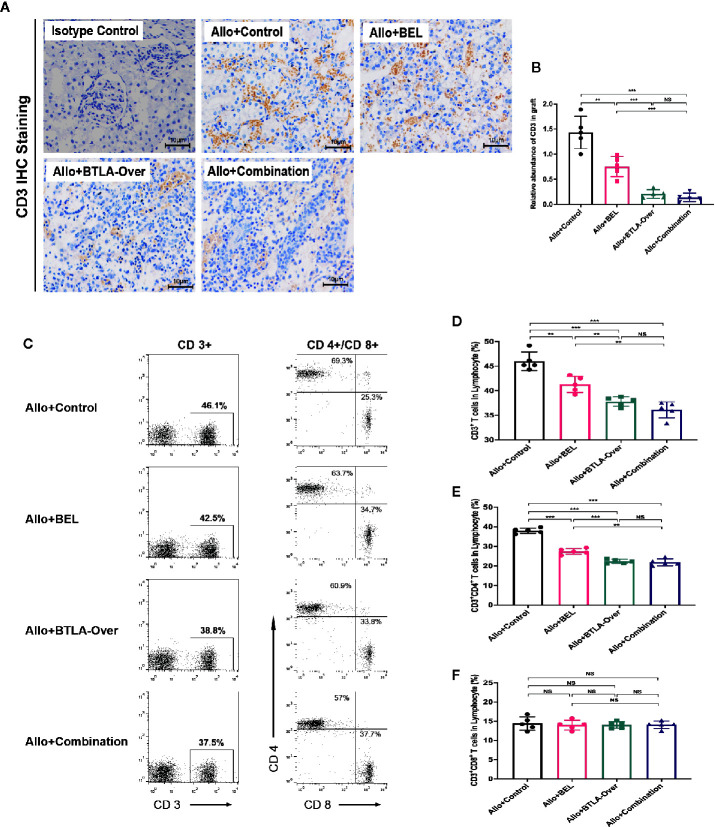
Combination therapy inhibited receptor peripheral T cells and reduced graft CD3+ cell infiltration. **(A)** Analysis of CD3+ cell infiltration in transplanted kidney tissue by immunohistochemical staining. Magnification: 400×. **(B)** Integral optical density value (IOD) was used to indicate the relative expression of CD3 in the tissue. **(C)** Flow cytometry to detect CD3, CD4, CD8 positive cell frequency in peripheral blood. CD4+/CD8+ cells were circled from CD3+ cells in total lymphocyte. **(D)** Percentage of CD3+ cells in total lymphocytes. **(E)** Percentage of CD3+CD4+ cells in each group to total lymphocytes. **(F)** Percentage of CD3+CD8+ cells to total lymphocytes in each group. Results are expressed as mean ± SD, NS, no significant; **P < 0.01; ***P < 0.001.

Both Belatacept and BTLA target the T cell surface coinhibitory molecules, which mainly affect the proliferation and activation of T cells. Therefore, we further observed T cell changes to explore the specific mechanism. Data from IHC staining indicated that the Allo+Control group had more CD3 infiltration in graft tissues, whereas both Belatacept and BTLA overexpressed reduced CD3 expression (**Figures 3A, B**). This result suggests that combination therapy suppressed T cell infiltration in the graft during the acute rejection period. To research the source of the reduction of T cell infiltration, we analyzed CD3, CD4 and CD8 positive cells in peripheral blood by flow cytometry (**Figure 3C**). We found that combined Belatacept and BTLA overexpression significantly reduced the composition of CD3+ T cells in total lymphocytes in the postoperative peripheral blood compared with the control group (**Figure 3D**).

At the same time, we observed the expression of CD4+ and CD8+ cells in CD3+ T cells. The inhibitory effect of targeted BTLA on CD3+CD4+T cells was stronger than that of Belatacept treatment alone and largely consistent with the combination treatment (**Figure 3E**). Notably, there was no significant difference in CD3+CD8+ T cell expression between these groups (**Figure 3F**). Taken together, these results indicate that the combined treatment mainly inhibited CD4+ T cells, rather than CD8+ T cells, in recipient peripheral blood lymphocytes after kidney transplantation to reduce the total number of CD3+ T cells and subsequently attenuate T cell infiltration in the grafts. Meanwhile, the inhibitory effect of BTLA overexpression on T cells was stronger than that of Belatacept.

### Belatacept Combined With BTLA Overexpression Inhibited T Lymphocyte Proliferation

**Figure 4:**
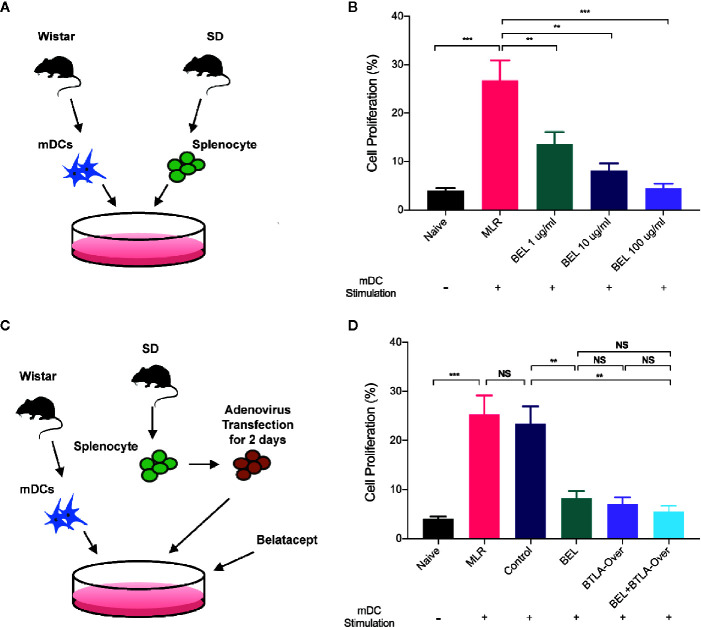
Belatacept combined with BTLA overexpression inhibited lymphocyte proliferation. **(A)** Schematic of the mixed lymphocyte reaction, extracting mature dendritic cells from Wistar rat as stimulus cells and SD rat spleen lymphocytes as receptor cells for mixed culture. **(B)** Proliferative changes in lymphocytes after different doses of Belatacept intervention by BrdU incorporation and flow cytometry analysis. **(C)** Schematic diagram of combination treatment in mixed lymphocyte cultures. **(D)** The proliferation of receptor lymphocytes under single versus combination intervention. Results are expressed as mean ± SD, NS: no significant; **P < 0.01; ***P<0.001.

We conducted MLR to stimulate antigen-specific immune response *in vitro* to initially test the effects of Belatacept and BTLA on cell proliferation. The BrdU positive rate in response cells was detected by flow cytometry at 3 days of MLR to reflect the change of proliferation (**Figure 4A**). The results, as shown in **Figure 4B**, suggest that compared with normal splenocytes (the Naïve group), the cell proliferation rate of the MLR group was significantly higher after stimulation. Furthermore, Belatacept inhibited T lymphocyte proliferation in a dose-dependent manner.

Western blot and qRT-PCR analyses were used to verify the transfection efficiency of BTLA adenovirus (**Figure S4**). Then, recipient cells were pretransfected with BTLA-overexpression adenovirus 2 days prior to MLR and intervened with 10 ug/ml Belatacept in the combined treatment group (**Figure 4C**). No significant difference in cell proliferation was found between the negative vectors in the Control group and the MLR group. Compared to the Control group, lymphocyte proliferation was significantly inhibited by Belatacept combined with BTLA overexpression (the BEL+BTLA-Over group) (**Figure 4D**). These results suggest that Belatacept, overexpression of BTLA, and combination therapy inhibited lymphocyte proliferation *in vitro*.

### Belatacept and BTLA Overexpression Combination Affected Cytokine Production

**Figure 5:**
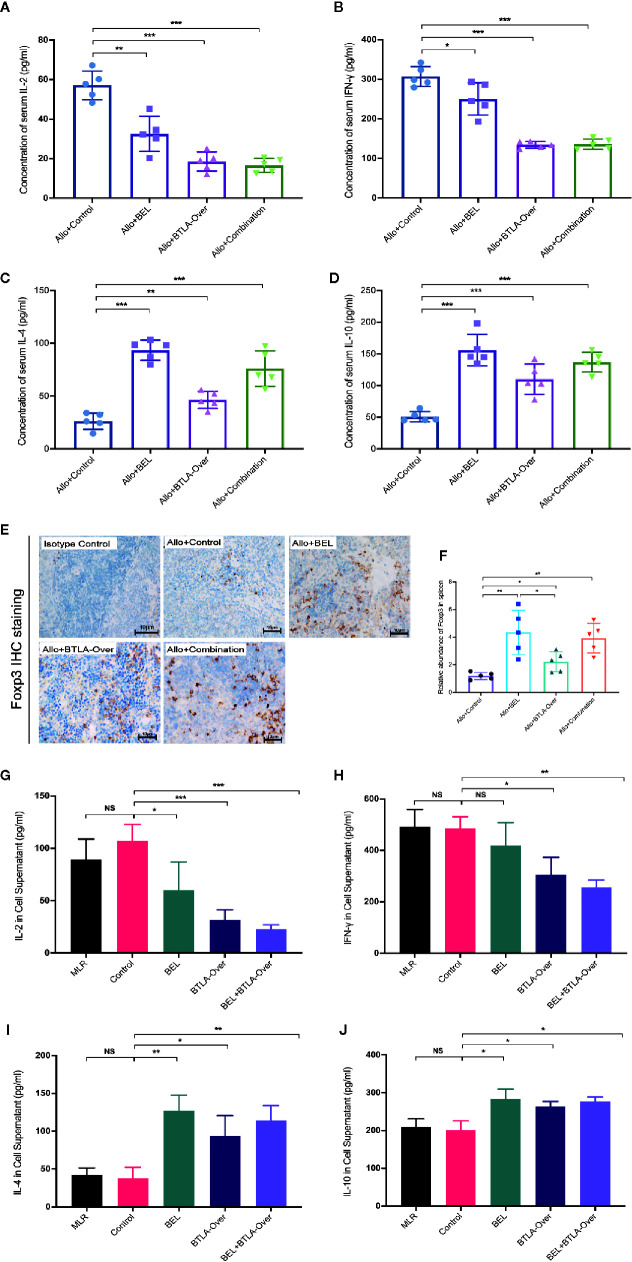
Belatacept combined with BTLA overexpression affected T cell differentiation in immune response. **(A–D)** ELISA test for serum cytokine expression levels in recipient rats on day 7 after kidney transplantation. (**A**: IL-2; B: IFN-γ; C: IL-4; D: IL-10). **(E)** Immunohistochemical staining analysis of Foxp3 in recipient spleen tissues on day 7 after surgery. Magnification: 400×. **(F)** Integral optical density value (IOD) was used to indicate the relative expression of Foxp3 in spleen tissue. **(G**–**J)** ELISA was used to detect cytokine expression in supernatants of mixed lymphocytes after 72 h of culture. (**G**: IL-2; H: IFN-γ; I: IL-4; J: IL-10). Results are expressed as mean ± SD, NS, no significant; *P < 0.05; **P < 0.01; ***P < 0.001.

ELISA was performed to examine the expression of cytokines IL-2, IFN-γ. IL-4 and IL-10. In an *in vivo* experiment, the results showed that serum IL-2 and IFN-γ expression were significantly decreased in the Allo+Combination group compared to the Allo+Control group. In addition, Belatacept treatment upregulated IL-4 and IL-10 levels compared with negative control, more significantly than BTLA overexpression, and combination treatment similarly stimulated IL-4 and IL-10 secretion. These results indicate that combined therapy inhibited the secretion of serum IL-2 and IFN-γ, as well as induced the production of IL-4 and IL-10 (**Figures 5A–D**). According to Foxp3 IHC analysis of postoperative spleen tissues, we found that Belatacept combined with BTLA overexpressed significantly upregulated Foxp3 expression in spleen tissues compared to the control group (**Figures 5E, F**).

Subsequent analysis was done in MLR to examine the expression of cytokines in the supernatant after 72 h *in vitro* (**Figures 5G–J**). The Control group pretransfected with adenovirus negative vectors did not differ significantly from the normal MLR group in terms of individual cytokine expression. Similar to the *in vivo* results, the cointervention of BEL+BTLA-Over significantly inhibited the secretion of IL-2 and IFN-γ in response cells but upregulated the expression levels of IL-4 and IL-10 in the supernatant compared with the Control group (P<0.05). Interestingly, there was no significant difference of IFN-γ in the BEL group compared with the Control group, and Belatacept stimulated the secretion of IL-4 and IL-10. The above experimental results show that Belatacept and BTLA overexpression changed cytokine expression levels, suggesting possible changes in the differentiation of T cells secreting these cytokines.

### CTLA-4 and BTLA Expression Upregulation in Antigen-Specific Immune Responses Differed Over Time

**Figure 6:**
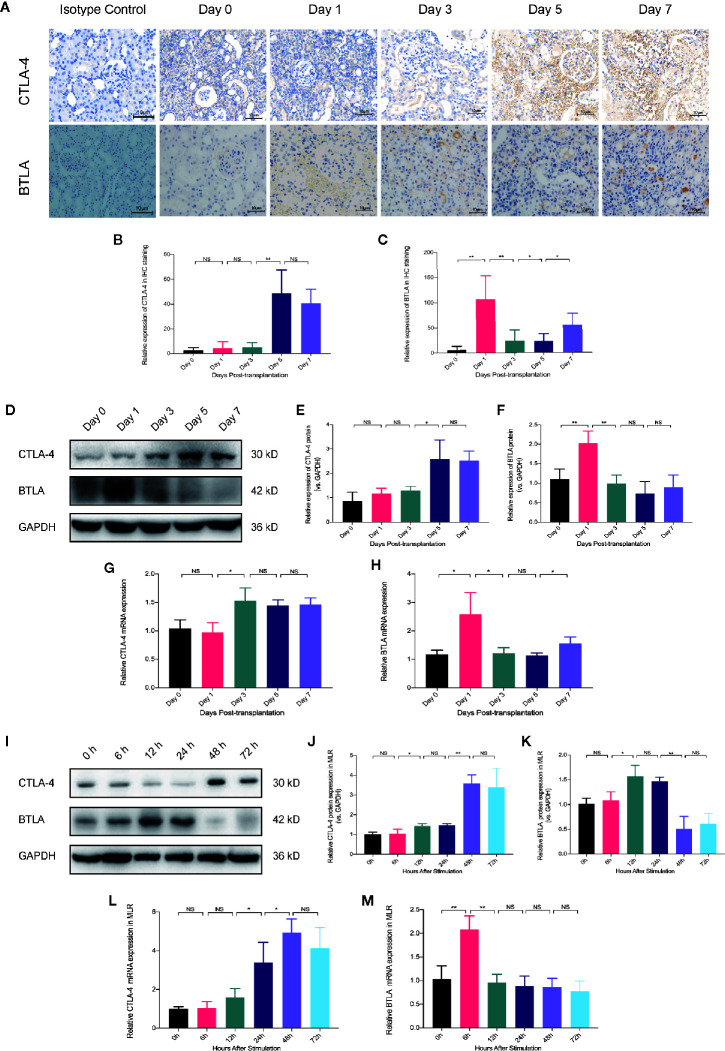
The timing of CTLA-4 and BTLA expression upregulation in antigen-specific immune responses is different. **(A)** Immunohistochemical staining analysis of CTLA-4 and BTLA in renal tissues at different postoperative time points. Magnification: 400×. **(B)** Analysis of relative expression of CTLA-4 in the grafted kidney by IOD values. **(C)** Relative expression of BTLA in immunohistochemical staining. **(D)** Expression levels of CTLA-4 and BTLA in total graft protein from recipients at different postoperative time-points were analyzed by western blot. **(E)** Relative expression of CTLA-4 protein in kidney graft. **(F)** Relative expression of BTLA in total graft protein. **(G)** Analysis of relative expression of CTLA-4 mRNA in the postoperative recipient at different time points by qRT-PCT. **(H)** Relative expression levels of BTLA mRNA in transplanted kidney tissue. **(I)** Western blot analysis of CTLA-4 and BTLA protein expression in mixed lymphocytes reaction (MLR) after 0, 6, 12, 24, 48, and 72 h. **(J)** Analysis of relative expression of CTLA-4 protein in MLR receptor cells. **(K)** Analysis of relative BTLA protein expression in receptor lymphocytes. **(L)** qRT-PCR analysis was used to detect the relative expression of CTLA-4 mRNA in MLR at different times. **(M)** qRT-PCR analysis was used to detect the relative expression level of BTLA mRNA. Results are expressed as mean ± SD, NS, no significant; *P < 0.05; **P < 0.01.

To explore the differences in the effects of Belatacept and overexpressed BTLA along with the mechanisms of combined treatment on cell differentiation, we evaluated the expression of CTLA-4 and BTLA at different times in an *in vivo* model and in an *in vitro* model. IHC staining of grafts showed that CTLA-4 expression levels on postoperative days 1 to 3 were similar to those of the preoperative period; however, CTLA-4 infiltration increased in tissues on postoperative day 5, increasing more than 40-fold compared with preoperative day 0. BTLA expression was rapidly increased on day 1, then began to decrease on day 3, and by day 7 expression levels were lower than day 1 (**Figures 6A–C**). In addition, we extracted transplanted kidney protein and RNA and measured CTLA-4 and BTLA expression by western blot and qRT-PCR analyses, which matched the above results (**Figures 6D–H**). These findings indicate that both CTLA-4 and BTLA were upregulated in the early stages of acute rejection, and BTLA expression was increased earlier than CTLA-4.

Then, we examined the protein and mRNA expression of CTLA-4 and BTLA in receptor cells at 0, 6, 12, 24, 48, and 72 h after MLR to observe the time-trend (**Figures 6I–M**). We found that CTLA-4 upregulated mRNA expression at 24 h after stimulation and showed high protein expression at 48 h, while BTLA increased mRNA expression at 6 h and then began to decrease as well as upregulated BTLA protein at 12 h. These trends are similar to those of *in vivo* experiments and suggest that CTLA-4 and BTLA may act early in the immune response after antigenic stimulation, and BTLA expression was elevated earlier than CTLA-4.

## Discussion

Organ transplantation is the most effective treatment for organ failure, especially for the kidney (30). Nevertheless, the occurrence of acute rejection posttransplantation as a major reason for allogeneic graft dysfunction affects long-term survival, which calls for further exploration and optimization of immunosuppression treatment. Combined targeting of coinhibitory molecules provides a novel approach for the prevention and treatment of acute rejection. In the present study, we found that Belatacept combined with BTLA altered T cell differentiation, reduced DSA production, inhibited mixed rejection as well as prolonged graft survival.

We initiated antigen-specific immune responses *in vitro* through MLR and observed that both single and combination therapy effectively inhibited receptor cell proliferation. This finding suggests that Belatacept linked with BTLA overexpression reduced the degree of the alloimmune response and potentially functions to inhibit rejection after kidney transplantation. As previously described (31), we selected MHC fully mismatched Wistar and SD rats to construct a homozygous allogeneic kidney transplantation model to induce the onset of acute rejection *in vivo*. The allogeneic rats underwent a significant increase in postoperative serum creatinine, a dramatic loss of graft function and anuria around day 7. Allogeneic grafts showed worse pathological changes such as interstitial inflammation and tubulitis after transplantation, and by the seventh day, there was severe intimal arteritis and peritubular capillaritis, which revealed the coexistence of acute cell- and antibody-mediated rejection. Several studies have approved a correlation between C4d-positive staining, DSA, and histopathological findings in ABMR (32, 33), and the deposition of pericapillary complement C4d is highly suggestive of severe humoral rejection responses (34, 35). In this model, allogeneic recipients showed significantly elevated DSA on postoperative day 7, accompanied by C4d-positive staining with deposition of CD3-positive T cells in the transplanted kidney, which is similar to previous findings (36). These findings all point to the existence of severe mixed acute rejection.

As a maintenance drug, continued Belatacept administration contributes to the prevention and treatment of acute rejection and maintains long-term graft survival (37). In this study, we administered Belatacept twice postoperatively in rats, and the results showed that early noncontinuous administration failed to induce immune tolerance. Considering that adenovirus achieves stable expression 1–2 days after injection and can maintain its effect for approximately 7–10 days, we injected it 2 days preoperatively, resulting in BTLA overexpression. Although the single adenovirus treatment also failed to produce immune tolerance, the graft median survival time was prolonged to 14 days, which is similar to other findings of BTLA in organ transplantation (26, 38). As we surmised, after combination therapy, the recipients showed remarkable improvement in renal function and prolongation of graft survival time compared to the monotherapy, suggesting a more favorable effect of coadministration against early postoperative acute rejection and possibility of inducing immune tolerance. We performed further pathological analysis showing that mixed acute rejection was attenuated with a reduction of C4d deposition in the combined treatment group. Furthermore, combination therapy decreased the infiltration of CD138+ cells in grafts, accompanied by downregulation of circulating DSA production. Similar to our previous work (26), BTLA overexpression reduced the generation of intimal arteritis in the graft and effectively suppressed TCMR. Interestingly, we found that Belatacept had an inhibitory effect on the ABMR but a weaker therapeutic effect on TCMR such as arterial endarteritis than single targeting BTLA. There has been less research on the function of Belatacept in ABMR (39), and recent studies showed that it appears to block the CD28-mediated activation of T follicular helper cells (Tfhs), thereby modulating B cells and reducing DSA production (40). A primate modeling study also reported a disruption of the germinal center by Belatacept (41). In the clinic, *de novo* DSA development in the phase III BENEFIT and BENEFIT-EXT studies showed that Belatacept-based immunosuppression is associated with a significantly lower incidence of *de novo* DSA development relative to cyclosporine-based immunosuppression over 7 years (84 months) of follow-up (42). These findings are similar to those we found, where Belatacept had a potential inhibitory effect on ABMR.

By observing T cell expression, we found that the nondifference in CD8+ cells between experimental groups and the significant change in CD4+ cells suggested that the combination therapy exerts inhibition mainly through regulating CD4+ T cells, thereby significantly reducing the frequency of circulating CD3+ cells and thus the infiltration of CD3+ T cells in grafts. The obvious effect of BTLA on T cells also explains its potent inhibition of TCMR. Serum cytokine levels were detected to understand the altered CD4+ cell differentiation in circulation. During acute rejection, naïve CD4+ T cells, stimulated by donor antigens, mainly differentiate into Th1 and Th2 cells. Th1 cells participate in rejection by secreting the inflammatory cytokines IL-2 and IFN-γ, which can also respond to the degree of T cell immune response (43). Th2 cells that secrete IL-4 and IL-10 are thought to have a dual role of anti-inflammatory and rejection suppression and are involved in inducing immune tolerance (44, 45). In our study, we found that Belatacept inhibited the secretion of IL-2 and IFN-γ more weakly than targeting BTLA but increased IL-4 and IL-10 production. This finding also suggests that Belatacept probably reduces Th1 cells and increases Th2 cells, while BTLA overexpression probably significantly inhibits Th1 cells, thereby reducing the proportion of CD4+ T cells. The shift of Th1 cells to Th2 cells is known as one of the mechanisms for constructing graft immune tolerance (46). Based on the changes in cytokine expression levels, we speculate that the combination treatment may downregulate Th1 cells and increase Th2 cell differentiation, causing Th1/Th2 shifts with prolonged graft survival. In addition, Foxp3 acts as a marker for regulatory T cells (Tregs) and has a protective effect on infiltration in immune organs. Foxp3+ T follicular regulatory cells (Tfrs) have recently been found to inhibit the onset of ABMR promoted by Tfh cells (47, 48). The upregulation of splenic Foxp3 in the Allo+Combination group also revealed a possible alteration in regulatory T cell differentiation. Therefore, Belatacept combined with BTLA overexpression may alter CD4+ T cell differentiation, affect the Th1/Th2 cell shift, promote regulatory T cell production, and thus inhibit acute rejection after renal transplantation.

After naïve CD4+ T cells are stimulated with antigen, Th1 differentiation predominates in the early stages, followed by Th2 cells that begin to secrete cytokines and exert inhibitory effects (49, 50). Similar to other studies, CTLA-4 expression began to increase after 24–48 h of antigenic stimulation *in vitro*, whereas BTLA expression was stimulated in cell culture at 6–12 h. In acute rejection after kidney transplantation, high expression of CTLA-4 was observed from postoperative day 5. The expression of BTLA is increased on postoperative day 1 and then rapidly decreases. These results suggested that BTLA acts as an early indicator of acute rejection and may exert its inhibitory effects earlier than CTLA-4. Based on these observations, we speculated that BTLA acts early in the acute rejection, possibly by effectively inhibiting Th1 cell differentiation, suppressing T cell proliferation and exhibiting a significant anti-TCMR effect. CTLA-4 was elevated later than BTLA, manifesting as inhibition of Th1 cells and stimulation of Th2 cell differentiation. This also revealed that different costimulatory molecules may have different action times in the antigen-stimulated immune response, with different mechanisms of action. Combination therapy has a better inhibitory effect on early acute rejection than single therapy.

Considering the potent effect of overexpressed BTLA on TCMR, it is possible that Belatacept provided a synergistic inhibitory effect, which offers hope for an immunosuppressive regimen free of CNIs. However, this still needs to be further verified in future comparative studies. Additionally, combination therapy effectively suppressed mixed rejection and can improve graft survival in patients with a risk of poor prognosis due to misdiagnosis, as TCMR or ABMR alone is an inadequate immunosuppressive therapy (51, 52). Combination therapy significantly prolonged the immunosuppressive state, suggesting the potential benefit of reducing the immunosuppressive dosage, minimizing drug toxicity and reducing the incidence of adverse events.

## Conclusion

Overall, in both *in vivo* and *in vitro* experiments, we found that Belatacept reduced the production of DSA and had a probable inhibitory effect on acute ABMR after kidney transplantation. Belatacept combined with BTLA overexpression prolonged graft survival possibly by regulating circulating T cell differentiation, causing a Th1/Th2 cell shift, reducing T cell and plasma cell infiltration and inhibiting acute rejection. CTLA-4 and BTLA may explain the different effects of targeted therapy on T cell differentiation by their different durations of action in the immune response. In brief, Belatacept combined with BTLA overexpression can attenuate acute rejection after kidney transplantation and prolong graft survival, which provides new ideas for the optimization of early immunosuppression protocols after clinical renal transplantation.

## Data Availability Statement

The original contributions presented in the study are included in the article/**Supplementary Material**. Further inquiries can be directed to the corresponding authors.

## Ethics Statement

The animal study was reviewed and approved by the Animal Care and Use Committee of Nanjing Medical University.

## Author Contributions

MG, HZ, and ZW conceived and designed this study. HZ, JZ, ZG, and HY performed the experiments and collected the data. HZ, ZW, JZ, HC, RT, and AC completed the data analysis and interpretation. AC provided essential suggestions for the data presentation. All authors contributed to the article and approved the submitted version.

## Funding

This work was supported by the National Natural Science Foundation of China (grant numbers 82070769, 81900684, 81870512, 81770751, 81570676, 81470981, and 81100532), Project of Jiangsu Province for Important Medical Talent (grant number ZDRCA2016025), the “333 High Level Talents Project” in Jiangsu Province (grant numbers BRA2017532, BRA2016514, and BRA2015469), the Standardized Diagnosis and Treatment Research Program of Key Diseases in Jiangsu Province (grant number BE2016791), the Open Project Program of Health Department of Jiangsu Province (grant number JSY-2-2016-099), and the Jiangsu Province Natural Science Foundation Program (grant number BK20191063).

## Conflict of Interest

The authors declare that the research was conducted in the absence of any commercial or financial relationships that could be construed as a potential conflict of interest.
