# Supplementary material for: Combined Immunotherapy With Belatacept and BTLA Overexpression Attenuates Acute Rejection Following Kidney Transplantation
Source: Front Immunol. 2021 Feb 24;12:618737. doi: 10.3389/fimmu.2021.618737 (PMC7959759; doi:10.3389/fimmu.2021.618737)
Supplement: Supplementary file 1 [file DataSheet_1.pdf]

Table S1:

Group	Recipient	Donor	Cold-ischemic time	Surgery time	Surgical success rate*
Syn	SD	SD	32 ± 3.4 mins	94 ± 5.1 mins	96%
Allo	SD	Wistar	35 ± 3 mins	105 ± 6.5 mins	93%
Allo+Control	SD	Wistar	38 ± 4.4 mins	110 ± 4.2 mins	89%
Allo+BEL	SD	Wistar	35 ± 2.6 mins	109 ± 8.6 mins	93%
Allo+BTLA-Over	SD	Wistar	37 ± 4.7mins	113 ±10.4 mins	96%
Allo+Combination	SD	Wistar	36 ± 2.8 mins	111 ± 6.9 mins	93%

Table S1. Surgery-related data for each group.

All values are expressed as mean ± SD. *: Surgical success means anastomosed renal arteriovenous of recipients filling successfully after reperfusion and fully recovered from anesthesia.

Figure S1:

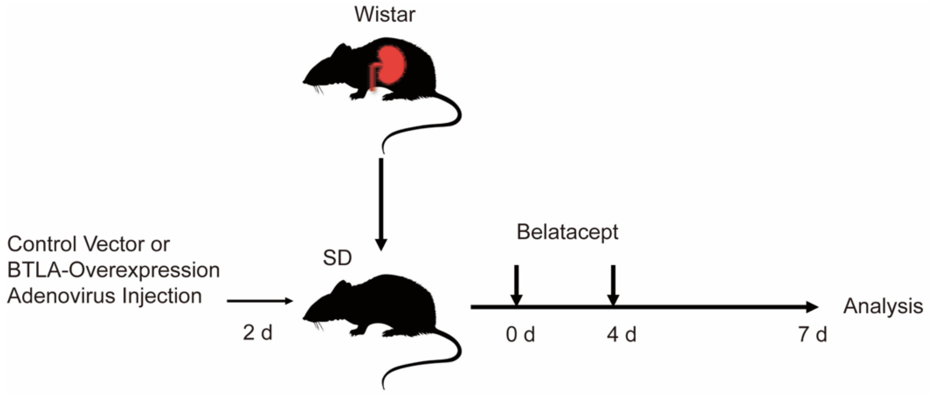

Figure S1. Schematic of the combined treatment regimen for rats in the Allo+Combination group.

Figure S2:

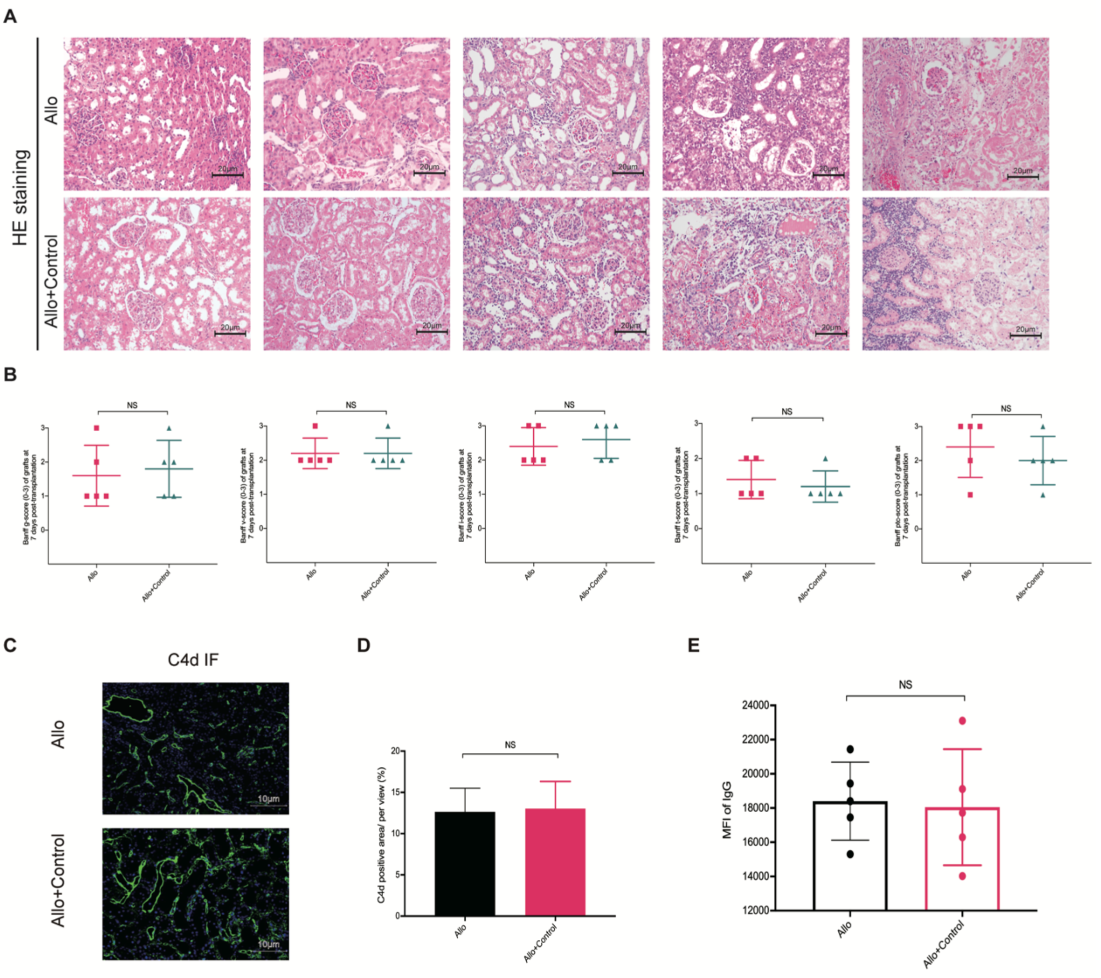

Figure S2. Compare the acute rejection with or without negative-control vectors in Allogeneic recipients.

(A) Pathological staining analysis of kidney grafts from the Allo group and the Allo+Control group recipients on preoperative Day 0 and postoperative Days 1, 3, 5 and 7 (Magnification: 200×). (B) Assessment of graft tissues glomerulitis (g), intimal arteritis (v), interstitial inflammation (i), tubulitis (t), and peritubular capillaritis (PTC) based on the Banff 2017 classification system. (C) Graft tissue-specific C4d immunofluorescence staining analysis on 7 days after transplantation (Magnification: 400×). (D) The proportion of C4d-positive regions was used to compare relative C4d-positive expression across groups. (E) Expression of serum DSA were reflected by mean fluorescence intensity (MFI) of donor-related IgG in flow cytometry analysis. Results are expressed as mean ± SD, NS: no significant.

Figure S3:

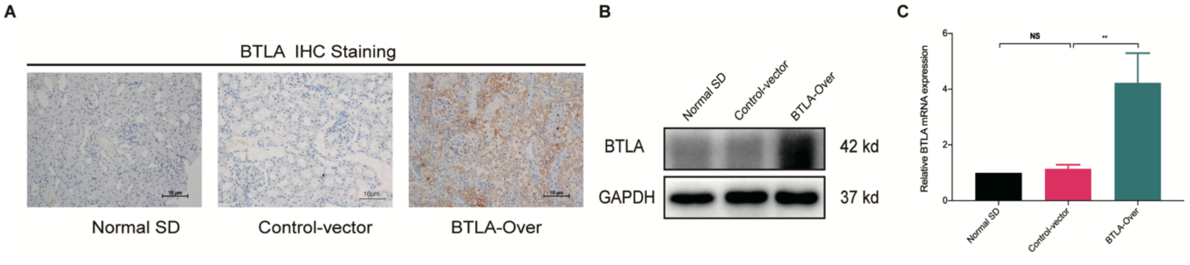

Figure S3. The validation of BTLA overexpression adenovirus transfection in vivo at the normal SD rats.

(A) Immunohistochemical staining analysis of BTLA expression in kidney tissues of normal SD rats, adenovirus negative vectors and overexpression adenovirus 2 days after intravenous injection. (B) Western blot analysis of BTLA protein expression levels in kidney tissues from each treatment group. (C) Relative expression of BTLA mRNA in renal tissues 2 days after transfection in each group. Results are expressed as mean ± SD, NS: no significant; **: P<0.01.

Figure S4:

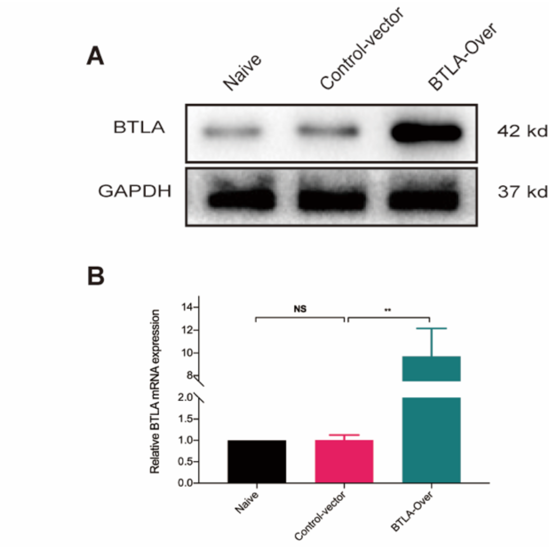

Figure S4. The validation of BTLA overexpression adenovirus transfection in response lymphocyte.

(A) Western blot analysis of BTLA protein expression in spleen lymphocytes of untreated, adenovirus negative vector transfected and BTLA overexpression adenovirus transfected 48 hours after transfection. (B) qRT-PCR analysis for relative BTLA mRNA expression 48 hours after transfection in each group. Results are expressed as mean ± SD, NS: no significant; **: P<0.01.
